# Emotional dysregulation subgroups in patients with adult Attention-Deficit/Hyperactivity Disorder (ADHD): a cluster analytic approach

**DOI:** 10.1038/s41598-019-42018-y

**Published:** 2019-04-04

**Authors:** Oliver Hirsch, Mira Lynn Chavanon, Hanna Christiansen

**Affiliations:** 1 FOM University of Applied Sciences, Siegen, Germany; 2 Department of Clinical Child and Adolescent Psychology and Psychotherapy, Philipps University Marburg, Marburg, Germany

## Abstract

Emotion regulation deficits (ERD) are evident in about 34–70% of the adults with ADHD. In contrast to this, they are not considered in the diagnostic criteria of the disorder. In a recent study of our research group using confirmatory factor analysis, we modeled positive and negative emotion as well as emotion regulation skills along with the classical ADHD-core symptoms. We showed that negative affect and the failure to apply adaptive emotion regulation skills were distinct and indicative dimensions in adult ADHD. In this study, we used a person-centered approach based on cluster analysis to subtype patients on the presence or relative absence of ERD. This results in important information to individualize treatment decisions. We found two clusters, with cluster 2 showing high ERD that were associated with higher impairments indicated by depressive mood, negative affect and elevated psychological distress. There were also higher rates of comorbidity in cluster 2 such as somatoform disorders which were associated with ERD. Women were overrepresented in this cluster 2. Neuropsychological variables did not contribute significantly to cluster formation. In conclusion, ADHD in adults is a heterogeneous disorder with specific subgroups that need differential treatment approaches.

## Introduction

Emotions are temporally limited, qualitative states that are associated with a change on the level of feelings, expression and physical states^1^. Emotion generation can be described in four steps: (1) an emotional relevant situation occurs; (2) we direct our attention to this situation; (3) we judge and evaluate the situation which results (4) in an emotional reaction, that can then either be regulated or not^2^. A common model for emotion regulation (ER) is the one by Gross^3^. According to this model, emotions that need regulating have to be identified; an ER strategy has to be picked; and this strategy has to be implemented and evaluated whether the strategy is sufficient or needs adaptation. ER are thus all processes that enable us to influence our emotions in such a way as to when and how we experience and express them.

ER is proposed as an additional sixth factor within the Research Domain Criteria Initiative (RDoc) of the National Institute of Mental Health (NIMH) that reflects interactions among the other five domains^4,5^. Recent studies in the field have shown that 40% to 70% of mental disorders are characterized by emotion dysregulation – with Attention Deficit/Hyperactivity Disorder (ADHD) being one of them^6,7^. This line of research points to ER and dysregulation as a biologically founded, dimensional, key transdiagnostic factor cutting across the specified R-DoC domains of the negative (ie, responses to threat/fear) and positive valence systems (ie, responses to reward), the cognitive system (ie, regulation of capacity limited systems such as awareness, higher perceptual processes, motor action), and system of social processes (ie, affiliation/attachment and social communication) as well as the arousal/regulatory system (ie, activation of neural systems for various contexts)^8^. As such, ER deficits distinguish between groups of healthy and mentally ill people, and are basically observable for all mental disorders with experimental research^9^, demonstrating that such deficits are central to the development and maintenance of psychopathology^10,11^.

In children, adolescents and adults with ADHD common co-occurring features relate to altered emotional experience like irritability, emotional hyper-responsiveness, rapidly changing moods and impaired emotion regulation as seen in reactive aggression, temper outbursts and poorly controlled behavior in emotionally colored situations^12–14^. A comprehensive review on ADHD and ER concluded that emotion regulation deficits (ERD) are evident in about 34–70% of adults with ADHD^15^. Those features are associated with mental health problems and unfavorable psychosocial outcomes in the mid- and long-term^16–18^. For instance, high levels of emotional lability explain a substantial portion of variance related to the morbidity and burden of adult ADHD^12^, and ERD was associated with significant functional impairments such as professional and familial instability^17,19^, as well as high-risk behaviors, such as risky sexual or driving behaviors^19,20^. Accordingly, ERD has been recognized as an important dimension of ADHD^15,21,22^, but is neither considered in the diagnostic criteria for the disorder in the Diagnostic and Statistical Manual of Mental Disorders^23,24^ nor in the International Classification of Diseases 10th revision^25^. Reasons why ERD had not been included as a third dimension aside attention deficits and hyperactivity/impulsivity are that those symptoms are not specific to ADHD^26,27^, vary substantially even in healthy controls^28^, and finally an explanation for the etiology of those affective symptoms in ADHD as well as a clear-cut definition of the various emotional symptoms^29^ is missing. The latter is also recognized in the current practitioner review on emotional dysregulation in ADHD^29^. Though there is no single, standard definition of ERD, it is generally regarded as a multidimensional construct entailing a lack of inhibition together with strong negative and positive emotions, and the failure to engage in self-regulatory actions^27^. In the review by Shaw and colleagues^15^ three different models for the association between adult ADHD and emotion regulation that are also presented in the recent practitioner review^26^ are discussed.

The first model conceptualizes ERD as a core symptom of ADHD^30–32^ based on a joint neurocognitive deficit, in the way that emotion regulation deficits in ADHD are underpinned by broader aspects of self-regulation and executive control^33^. For the second model it is argued that ERD entails some specific and dissociable neurocognitive components beyond executive dysfunctions^34^, that is ERD and ADHD are seen as correlated but distinct dimensions with overlapping, though separable neurocognitive deficits. This is supported by recent observations that emotion regulation independently contributed to the distinction between children with ADHD and typically developing children^35,36^. The third model regarding the observed overlap between ADHD and emotional dysregulation emphasizes the need to consider the combination of ADHD and ERD as a separate entity^37^. This view is supported by the observation that emotional dysregulation is more often seen in the combined ADHD presentation with a higher symptom load and a poorer outcome^14,22,38,39^ and by studies demonstrating that ADHD and bipolar disorder often occur together and aggregate in families at higher-than-expected rates^40^. Surman *et al*.^37^ for example showed that deficient emotional self-regulation was only elevated in siblings of adult patients with ADHD and deficient emotional self-regulation. At least it seems that ERD should be viewed as a defining feature of more severe adult ADHD^14^.

In order to further illuminate the link between ERD and ADHD specified by those three conceptual positions, we recently used a confirmatory factor analysis approach^41^. We modeled positive and negative emotion as well as emotion regulation skills along with the classical ADHD-core symptoms. We showed that negative affect and the failure to apply adaptive emotion regulation skills were distinct and indicative dimensions in adult ADHD^41^. Although this result supports ERD to be of special importance to ADHD, it neglects the obvious clinical heterogeneity of emotional symptoms observed in adult ADHD samples. Following the work by Nigg and colleagues^42^ on subtyping executive deficits in ADHD, the present study now uses a person-centered approach to model ADHD heterogeneity within the emotional dimensions we already established.

## Results

The k means cluster analysis algorithm within ALMO 15 has the possibility to examine the classification variables regarding their influence for cluster formation. Table 1 displays the partial η^2^ for the variables entered into the k means cluster analysis performed in ALMO 15.

**Table 1:** Partial η^2^ for the variables entered into the k means cluster analysis performed in ALMO 15.

Variable	η^2^
**Neuropsychological Performance**	
ASTM	0.023
Qb+	
Activity	0.033
Impulsivity	0.008
Inattention	0.017
**ADHD Core Symptoms**	
CAARS-S	
Inattention/Memory Problems	0.077
Hyperactivity/Restlessness	0.087
Impulsivity/Emotional Lability	0.274
Problems with Self-Concept	0.270
CAARS-O	
Inattention/Memory Problems	0.033
Hyperactivity/Restlessness	0.067
Impulsivity/Emotional Lability	0.132
Problems with Self-Concept	0.132
**Emotional Symptoms and Adaptive Emotion Regulation Skills**	
BDI	0.365
SCL-90-R GSI	0.373
EMO-Check	
Positive Affect	0.205
Negative Affect	0.397
ERSQ	0.196
**Personality**	
SCID-II	
self-defeating	0.244
dependent	0.239
obsessive-compulsive	0.052
negativistic	0.339
depressive	0.453
paranoid	0.282
histrionic	0.045
narcissistic	0.180
borderline	0.412
antisocial	0.055

ASTM = Amsterdam Short Term Memory Test, Qb = Quantified Behavior Test Plus, CAARS-S = Conners Adult ADHD Rating Scales self rating, CAARS-O = Conners Adult ADHD Rating Scales observer rating, BDI = Beck Depression Inventory, SCL-90-R GSI = Symptom Check List Global Severity Index, EMO-Check = EMO-Check Questionnaire, ERSQ = Emotion Regulation Skills Questionnaire, SCID-II = Structured Clinical Interview for DSM-IV.

A two-cluster solution with an F value of 87.21 and an η^2^ of 0.185 was proposed, meaning that 18.5% of the variance can be explained by this partitioning. This solution possesses good quality criteria^43,44^. It can be seen in Table 1 that several variables have small partial η^2^ values under the cut-off value for a medium effect^45^ of 0.06 and therefore contribute less to cluster formation. We therefore decided to exclude them from further analyses to obtain a stable cluster solution. Excluded were thus all neuropsychological variables ASTM, Qb+ Activity, Qb+ Impulsivity, Qb+ Inattention. Furthermore, we excluded CAARS-O inattention, and the SCID-II total number of symptoms regarding obsessive-compulsive, histrionic, and antisocial personality disorder.

**Figure 1:**
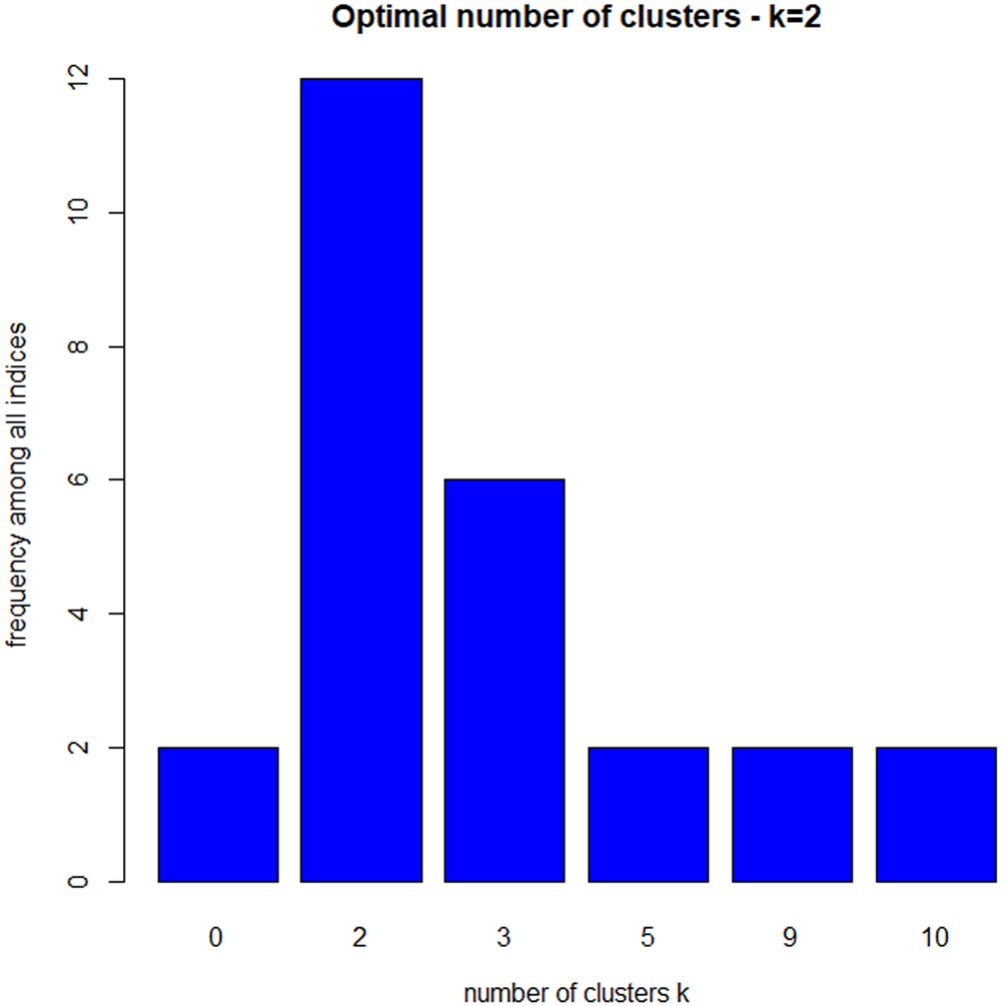
Optimal number of clusters in k means determined by R package NbClust.

We substantiated our analysis in R. Several indicators like average proportion of non-overlap (APN), average distance between means (ADM), figure of merit (FOM), Dunn index and Silhouette coefficient confirmed that the k means algorithm with two clusters should be the most appropriate grouping for our data (R package clValid)^46^. Within the R package NbClust, which contains 30 indices for choosing the best number of clusters, we further elaborated the optimal number of clusters in k means. The results are displayed in Fig. 1.

It can be seen in Fig. 1 that 12 indices propose two as the optimal number of clusters and six indices three clusters as the optimal number. We therefore chose the two-cluster solution. The ratio of the between sum of squares and the total sum of squares indicated that 25.0% of the variance can be explained by this partitioning. This approximately corresponds to the result in ALMO and also signals a satisfactory solution. Cluster 1 consisted of 181 (47%) and cluster 2 consisted of 204 (53%) patients.

The descriptive statistics of the classification variables in Table 2 reveal that subjects in cluster 2 have a higher symptom load on the CAARS: S & O, higher mean depressive symptoms (BDI-II), a higher total mean on the SCL-90-R, less positive affect, higher negative affect, larger difficulties in emotion regulation, and a higher symptom load on all included personality disorders of the SCID-II questionnaire. According to Cohen’s d effect sizes, most of the differences can be considered as large. All differences between the two clusters reach statistical significance (Welch-test, p < 0.0001) after adjusting for multiple testing.

**Table 2:** Descriptive statistics of classification variables used in k means cluster analysis.

	Cluster 1 (n = 181)		Cluster 2 (n = 204)		Cohen’s d	Welch-test
	Mean	SD	Mean	SD		
CAARS-S						
Inattention/Memory Problems	21.96	6.27	25.27	6.06	0.54	p < 0.0001
Hyperactivity/Restlessness	18.33	7.47	22.61	7.15	0.59	p < 0.0001
Impulsivity/Emotional Lability	17.51	6.00	24.97	5.52	1.30	p < 0.0001
Problems with Self-Concept	9.51	4.42	13.99	3.24	1.17	p < 0.0001
CAARS-O						
Hyperactivity/Restlessness	15.13	7.23	19.05	7.91	0.51	p < 0.0001
Impulsivity/Emotional Lability	16.08	7.10	21.98	7.87	0.79	p < 0.0001
Problems with Self-Concept	9.30	4.32	12.42	3.98	0.75	p < 0.0001
BDI	12.22	8.69	27.23	11.43	1.47	p < 0.0001
EMO-Check						
Positive Affect	46.27	18.52	28.22	16.97	1.02	p < 0.0001
Negative Affect	25.89	14.22	49.41	14.98	1.61	p < 0.0001
ERSQ	62.53	19.20	44.48	17.07	1.00	p < 0.0001
SCL90R GSI	0.66	0.39	1.45	0.59	1.57	p < 0.0001
SCID-II						
self-defeating	2.49 M: 2.48	1.92	4.56	1.81	1.11	p < 0.0001
dependent	2.65 M: 2.51	1.73	4.57	1.59	1.16	p < 0.0001
negativistic	2.88 M: 2.91	1.65	5.29	1.57	1.51	p < 0.0001
depressive	2.69 M: 2.69	1.90	5.81	1.60	1.79	p < 0.0001
paranoid	2.24 M: 2.09	1.78	4.70	1.93	1.33	p < 0.0001
narcissistic	2.71 M: 2.41	2.23	5.48 M: 5.16	3.46	0.94	p < 0.0001
borderline	4.77 M: 4.59	3.14	9.57	2.57	1.69	p < 0.0001

M = Huber’s M estimator. Welch tests and effect sizes Cohen’s d were calculated to assess differences between clusters. The significance value was adjusted for multiple testing: p = 0.05/19 = 0.0026.

In order, to get a better understanding of the ERSQ differences between the two clusters, we compared our data to previously published clinical and healthy samples (Table 3). Both, cluster 1 and cluster 2 report significantly less emotion regulation skills than two independent samples of healthy controls. The effect sizes for cluster 1 show that compared to healthy samples the deficits in ER are small to medium. For cluster 2, the same comparison reveals large deficits. Compared to clinical samples, cluster 2 even shows larger deficits than patients with major depressive and adjustment disorders, but similar deficits as patients with chronic recurrent depressive disorders. In contrast, cluster 1 reports better skills compared to other clinical groups.

**Table 3:** Descriptive Statistics of the ERSQ total mean score for cluster 1 and cluster 2 and previous, large samples using the ERSQ for assessing emotion regulation skills; low values indicate reduced skills.

	Cluster 1	Cluster 2	Lukas *et al*.^48^ HC	Grant *et al*.^49^ HC	Berking & Znoj^50^ HC	Lukas *et al*.^48^ AD	Lukas *et al*.^48^ MDD-SE	Lukas *et al*.^48^ MDD-RE
Mean	2.32	1.65	2.71	2.53	2.71	2.17	1.98	1.64
SD	0.71	0.63	0.52	0.68	0.52	0.72	0.79	0.71
N	181	204	214	263	576	477	444	421
Cluster 1	—	—	d = 0.64 [0.44, 0.84]	d = 0.31 [0.12, 0.5]	d = 0.68 [0.51, 0.85]	d = −0.20 [−0.38, −0.03]	d = −0.44 [−0.61, −0.26]	d = −0.95 [−1.13, −0.77]
Cluster 2	—	—	d = 1.84 [1.61–2.07]	d = 1.34 [1.14–1.54]	d = 1.87 [1.69–2.06]	d = 0.75 [0.58–0.92]	d = 0.45 [0.28–0.62]	d = −0.01 [−0.18–0.16]

Based on the reported mean and SD effect sizes Cohen’s d [CI95] was calculated to assess differences between both clusters with healthy and clinical comparison samples. For comparison with previous research, we report here the mean total score instead of the sum. HC = healthy controls, AD = Adjustment Disorder; MDD‐SE = Major depressive disorder, single episode; MDD‐RE = Major depressive disorder, recurrent episode; M = mean; SD = standard deviation.

The variables not used for classification in Table 4 show that the two clusters do not differ much regarding age and neuropsychological measures. The means in the CAARS DSM scales underline the higher degree of ADHD symptoms in cluster 2. It can be seen in Table 3 that several differences calculated by the Welch-test reach statistical significance after adjusting for multiple testing although the respective effect sizes are small to medium which show that these differences are not of predominant clinical relevance.

**Table 4:** Descriptive statistics of variables not used for classification in final cluster analysis solution.

	Cluster 1 (n = 181)		Cluster (n = 204)		Cohen’s d	Welch-test
	Mean	SD	Mean	SD		
Age	32.66	9.96	32.22	9.89	0.04	0.67
ASTM total score	83.55	7.24	80.88	10.36	0.30	0.003
Qb+						
Activity	1.71	1.17	2.08	1.13	0.32	0.0021
Impulsivity	0.92	1.33	1.16	1.44	0.18	0.09
Inattention	1.04	1.29	1.32	1.28	0.22	0.04
SCID-II						
schizotypical	1.96	1.91	3.46	2.21	0.72	<0.0001
schizoid	1.51	1.27	2.30	1.43	0.58	<0.0001
obsessive-compulsive	4.68	1.80	5.48	1.66	0.46	<0.0001
histrionic	1.60	1.65	2.19	1.67	0.36	0.0005
antisocial	2.64	2.58	3.96	3.35	0.44	<0.0001
CAARS-S						
DSM Inattentive Symptoms	16.98	4.69	19.45	4.49	0.54	<0.0001
DSM Hyperactive-Impulsive Symptoms	11.52	5.49	15.78	5.58	0.77	<0.0001
DSM ADHD Symptoms total	28.50	7.90	35.23	8.47	0.82	<0.0001
DSM ADHD Index	20.61	4.97	26.01	4.58	1.13	<0.0001
CAARS-O						
DSM Inattentive Symptoms	14.93	5.36	16.93	5.72	0.36	0.0005
DSM Hyperactive-Impulsive Symptoms	10.00	5.59	13.13	6.26	0.53	<0.0001
DSM ADHD Symptoms total	24.93	8.81	30.06	10.94	0.51	<0.0001
DSM ADHD Index	18.13	5.52	22.77	6.33	0.78	<0.0001
Impulsivity/Emotional Lability	16.08	7.10	21.98	7.87	0.79	<0.0001

Welch tests and effect sizes Cohen’s d were calculated to assess differences between clusters. The significance value was adjusted for multiple testing: p = 0.05/19 = 0.0026.

In cluster 1 the proportion of males is 67.4% (n = 122) while it is 54.4% (n = 111) in cluster 2. There is an association between cluster membership and gender (χ^2^ = 6.78, df = 1, p = 0.009). The effect size Cramér V signals a small effect with 0.13^47^.

Table 5 indicates that the proportions of ADHD presentations differ substantially between the two clusters (χ^2^ = 30.61, df = 2, p < 0.001). The effect size Cramér V signals a small effect with 0.29. Cluster 2 is dominated by patients with the combined type (85.0%) while in cluster 1 there is also a substantial proportion of patients with the predominantly inattentive type (35.6%).

**Table 5:** Frequencies and proportions of ADHD presentations in the two-cluster solution.

	Cluster 1	Cluster 2
Predominantly inattentive	63 (35.6%)	28 (14.0%)
Predominantly hyperactive-impulsive	8 (4.5%)	2 (1.0%)
Combined type	106 (59.9%)	170 (85.0%)

The two clusters differed significantly regarding the number of comorbid diagnoses (Fisher’s Exact Test, p = 0.001). In cluster 2 there is a higher proportion of patients with 2 comorbid diagnoses demonstrating that patients in cluster 2 have a higher symptom load (see Table 6).

**Table 6:** Frequencies and proportions of comorbid diagnoses in the two-cluster solution.

Number of comorbid diagnoses	Cluster 1	Cluster 2
0	107 (59.1%)	92 (45.1%)
1	51 (28.2%)	52 (25.5%)
2	19 (10.5%)	47 (23.0%)
3	4 (2.2%)	11 (5.4%)
4	0 (0%)	2 (1.0%)

The single comorbid diagnoses differentiated for the two clusters are listed in Table 7.

**Table 7:** Frequencies and proportions of single comorbid diagnoses in the two-cluster solution.

	Cluster 1 (n = 181)	Cluster 2 (n = 204)	
F1 Psychoactive substance use	Yes: 20 (11.0%) No: 161 (89.0%)	Yes: 39 (19.1%) No: 165 (80.9%)	χ^2^ (1) = 4.81, p = 0.03, Cramér V = 0.11
F2 Schizophrenia and delusional disorders	Yes: 3 (1.7%) No: 178 (98.3%)	Yes: 2 (1.0%) No: 202 (99.0%)	Fisher’s Exact Test p = 0.67
F3 Affective disorders	Yes: 50 (27.6%) No: 131 (72.4%)	Yes: 84 (41.2%) No: 120 (58.8%)	χ^2^ (1) = 7.76, p = 0.01, Cramér V = 0.14
F4 Somatoform disorders	Yes: 19 (10.5%) No: 162 (89.5%)	Yes: 49 (24.0%) No: 155 (76.0%)	χ^2^ (1) = 12.06, p = 0.001, Cramér V = 0.18
F5 Behavioral syndromes associated with physical factors	Yes: 4 (2.2%) No: 177 (97.8%)	Yes: 8 (3.9%) No: 196 (96.1%)	χ^2^ (1) = 0.93, p = 0.34, Cramér V = 0.05
F6 Personality disorders	Yes: 2 (1.1%) No: 179 (98.9%)	Yes: 5 (2.5%) No: 199 (97.5%)	Fisher’s Exact Test p = 0.46
F7 Mental retardation	Yes: 1 (0.6%) No: 180 (99.4%)	Yes: 0 (0%) No: 204 (100%)	Fisher’s Exact Test p = 0.47
F8 Psychological developmental disorders	Yes: 2 (1.1%) No: 179 (98.9%)	Yes: 0 (0%) No: 204 (100%)	Fisher’s Exact Test p = 0.22

The significance value was adjusted for multiple testing: p = 0.05/8 = 0.00625.

After correcting for multiple testing, the only significant difference on single comorbid diagnoses between the two clusters could be observed in somatoform disorders with cluster 2 having a significantly higher proportion (24.0%) than cluster 1 (10.5%). The effect size Cramér V signals a small effect. Cluster 1 and 2 do not differ significantly regarding affective disorders after Bonferroni correction and the resulting Cramér V shows a weak association.

## Discussion

Empirically derived symptom profiles based on cluster analysis from adult patients with ADHD revealed two clusters. Compared to healthy samples^48–50^ both of them were less skilled in ER, but compared to cluster 1, patients from cluster 2 reported more severe lack of skills: adult patients with ADHD from cluster 2 had highest ratings of emotional lability and reported the lowest ER skills, thus representing a subgroup of ADHD with severe ERD. Even compared to other clinical groups^48^ cluster 2 reported more impaired ER skills than comparison samples of patients with major depressive and adjustment disorders. In contrast, the total ER skill competence in cluster 1 was significantly higher than in samples of depressive, recurrently depressive and adjustment disordered patients.

Further, the percentage of women was elevated in cluster 2^51^ and there was a predominance of the combined presentation of ADHD according to DSM with about 85%. In cluster 1 we found more heterogeneous presentations of ADHD with substantial proportions of the inattentive and the combined subtype. Replicating results of international studies^22,51,52^ patients with severe ERD in cluster 2 were found to have higher levels of emotional symptoms as indicated by their reduced positive affect, elevated BDI score and negative affect ratings. In line with previous findings, we found the severe ERD in cluster 2 to be associated with higher impairments in most clinical areas as indicated by elevated SCL-90 GSI scores and heightened prevalence of somatoform disorders, substance abuse disorders and affective disorders^22^. Furthermore, the severe ERD cluster showed more comorbidities. Those results are in support of Shaw’s model^15^ three (ADHD + ERD as a distinct entity) in which the subgroup of ADHD + ERD is associated with higher symptom load, poorer long-term outcome and comorbidity. As the self-ratings also indicate higher comorbidity of cluster 2 with personality accentuations (see Table 4 SCID-II ratings), though those are not confirmed clinical disorders (see Table 7), future research should focus on the hypothesis that ERD may be linked to specific personality profiles^38^.

As our external validators are rather limited, we cannot shed light on the question whether our clusters differentially represent emotionally ill patients, patients with different levels of ER capacities or both. Although a pathway from ADHD through ERD to affective and temperamental liabilities and comorbidities seems plausible, there is a gap in the literature and our data cannot fill this gap. We are missing longitudinal data showing that severe ERD (as in cluster 2) compared to normal, impulsive ADHD (as in cluster 1) attracts further emotional symptoms and adverse outcomes like a magnet.

Surman *et al*. did not find neuropsychological tests that differentiated adults with ADHD and deficient emotional self-regulation from adults with ADHD without deficient self-regulation^52^. This corresponds to our findings showing that neuropsychological variables did not contribute significantly to cluster formation.

Most ADHD studies do not routinely assess emotional dysregulation. Therefore, it might be possible that these symptoms were misinterpreted as anxiety and/or depressive symptoms, especially in women with ADHD who have a higher rate of emotional dysregulation^51^. Recent data suggests that a substantial proportion of patients presenting with non-psychotic long-term mental health issues (e.g., depression, anxiety disorders, substance dependence disorders) fulfill a diagnosis of ADHD^53^.

Our cross-sectional data only represent the static view on ER and ERD and miss the dynamics central to emotion regulation^29^. As outlined in the introduction, emotion regulation processes include more than global self-reports of valence of emotional experience and intensity of emotions^3^. Thus, our analysis is just an attempt to organize ADHD subgroups based on self-reports regarding neurocognitive and emotional dimensions, but it is only one, static piece of the puzzle. Future research needs to address the dynamics, as understanding “emotion regulation in action” seems to be a prerequisite to tailor personalized treatment options: different problems in real-life emotion regulation (e.g. insensitivity to own emotions vs. insensitivity to context) will suggest different therapeutic goals and imply distinct interventions. Dialectical behavior therapy (DBT), an intervention that specifically targets emotional dysregulation, with modifications according to the special needs of patients with ADHD, has shown moderate to large effect sizes in treatments of adult ADHD, and might in light of our findings be worthy to be considered for further research^54–56^.

## Methods

### Participants

All participants were recruited from our specialized adult ADHD outpatient clinic (https://www.uni-marburg.de/de/fb04/team-christiansen/downloads/adulteadhs.pdf) based at the department of psychology at Philipps University Marburg, Germany. This clinic is specialized on diagnostics of adult ADHD and has a large catchment area. Our sample consisted of 385 individuals newly diagnosed with adult ADHD who were all medication-naive. They were examined by experienced licensed clinical psychologists on the basis of a detailed clinical history, the structured diagnostic interview for ADHD in adults (DIVA 2.0), a DSM-IV based clinical interview assessing the ADHD core symptoms in childhood and adulthood as well as psychological domains often impaired in adult ADHD^57^. Further, the Conners Adult ADHD Rating Scales (CAARS-L self- and observer-ratings), and the Qb+^©^ ^58^ were used for diagnostic assessments. Additionally, the Amsterdam Short Term Memory Test (ASTM) was applied as a symptom validity measure^59^. The diagnosis was based on the DIVA 2.0 results in order to fulfill DSM-IV diagnostic criteria.

Our sample consisted of 233 males (60.5%) aged a mean of 32.4 years (SD = 9.8) and 152 females (39.5%) aged a mean of 32.5 years (SD = 10.2). Twelve subjects (3.1%) had no school degree, 63 (16.4%) had basic schooling, 85 (22.1%) had finished secondary school, 199 (51.7%) had a grammar school (gymnasium) degree, and we had no information available from 26 patients (6.8%). Comorbidity was high in our sample, with 59 patients (15.3%) having a comorbidity in ICD-10 chapter V block F1 (psychoactive substance use), 5 patients (1.3%) in block F2 (schizophrenia and delusional disorders), 134 patients (34.8%) in block F3 (affective disorders), 68 patients (17.7%) in block F4 (somatoform disorders), 12 patients (3.1%) in block F5 (behavioral syndromes associated with physical factors), 7 patients (1.8%) in block F6 (personality disorders), 1 patient (0.3%) in block F7 (mental retardation) and 2 patients (0.5%) in block F8 (psychological developmental disorders). Summing up the number of comorbidities, 199 patients (51.7%) had no comorbidity, 103 patients (26.8%) one comorbidity, 66 patients two (17.1%), 15 patients three (3.9%), and 2 patients (0.5%) four comorbidities.

### Measures

#### Conners Adult ADHD Rating Scales (CAARS-L: S & O)

The German version of the CAARS-L: S assesses ADHD symptoms in adults aged 18 years or older. Symptoms are rated on a Likert-type scale (0 = *not at all*/*never* to 3 = *very much*/*very frequently*). The long version consists of 66 items that result in the four factors inattention/memory problems, hyperactivity/restlessness, impulsivity/emotional lability, and problems with self-concept. Confirmatory factor analyses of the German version in healthy adults and ADHD patients supported this factor analytic solution^60,61^. The four subscales are significantly influenced by age, gender, and the number of years of education. Symptom severity decreases with age, males score higher than females on hyperactivity and sensation-seeking behavior, and females score higher than males on problems with self-concept. Overall symptom ratings are higher for individuals with less education. Test-retest reliability ranges between 0.85 and 0.92, sensitivity and specificity are high for all four subscales. The CAARS-L: S represents a reliable and cross-culturally valid measure of current ADHD symptoms in adults^62^. The same holds true for the CAARS-L: O, the observer version, which comprises ratings on the same items by a person who has a close relationship to the subject under examination^63^. The hypothesized factor structure was supported and the observer version also possesses satisfactory psychometric properties.

#### EMO-Check

The EMO-Check Battery consists of two parts^64^. The first is a questionnaire for the self-assessment of therapy-relevant emotions, currently prepared for publication, that measures the extent of basic emotional states within the past week with 50 items on a five-point scale from “not at all” to “absolutely”. It is an extension of the Positive and Negative Affect Schedule (PANAS)^65^. It encompasses the ten items for positive and the ten items for negative affect, from the PANAS, adding additional items to cover emotions of stress, fear, anger, sadness, depression, and shame with 3 items each; guilt and disgust are measured with 1 item each. Furthermore, eleven items address the extent of coping emotions (optimism, courage, pride, etc.). In our study, we only used the two extended subscales for positive and negative affect with 25 items each.

The second part is the Emotion Regulation Skills Questionnaire (ERSQ)^50^ that assesses self-reports of adaptive responses to challenging feelings; it is based on the adaptive coping with emotions model^49,66^. The ERSQ is a 27-item self-report instrument employing a five-point Likert-type scale (0 = *not at all* to 4 = *almost always*) to assess these adaptive emotional regulation skills in the previous week. There are nine subscales containing three items each: *Awareness* (eg, “I paid attention to my feelings”), *Sensations* (eg, “My physical sensations were a good indication of how I was feeling”), *Clarity* (eg, “I was clear about what emotions I was experiencing”). *Understanding* (eg, “I was aware of why I felt how I felt”), *Acceptance* (eg, “I accepted my emotions”), *Tolerance* (eg, “I could endure my negative feelings”), *Readiness to confront distressing situations if needed to attain personally-relevant goals* (eg, “I pursued goals that were important to me, even if I thought that doing so would trigger or intensify negative feelings”), *Self-Support* (eg, “I supported myself in emotionally distressing situations”), and *Modification* (eg, “I was able to influence my negative feelings”). The total sum score was used for cluster analysis in the present study. When comparing our data to previously published data, we used the average score for ERSQ. As the ERSQ assesses ER skills as positive capacities, higher values indicate better skills, lower scores indicate deficient regulatory capacities. Internal consistencies are around 0.90, retest reliabilities range between 0.48 and 0.74. The total score correlated with the PANAS positive emotion subscale 0.41 and with the negative emotion subscale −0.33^50^. These data were confirmed in other studies^10,67^.

#### Beck Depression Inventory (BDI-II)

Depressive symptom severity was assessed with the revised Beck Depression Inventory^68,69^ which is a 21-item self-report measure assessing somatic, behavioral, emotional, and cognitive symptoms of depression on a 4-point scale ranging from 0 to 3. The total score ranges from 0–63, with scores higher than 14 points indicate clinically relevant levels of depressive symptoms. The German Version proved satisfactory internal consistency (α ≥ 0.84) and construct validity^70,71^.

#### Symptom Check List revised (SCL-90-R)

The SCL‐90‐R consists of 90 items (five-point Likert-scale ranging from 1 = not at all to 5 = very much) which assess nine primary symptom dimensions (somatization, obsessive–compulsive symptoms, interpersonal sensitivity, depression, anxiety, hostility, phobic anxiety, paranoid ideation, psychoticism). In addition, three global summary scores can be calculated. Reliability of the scales is satisfactory (α = 0.79 to α = 0.89) for subscales in clinical samples; to excellent (α = 0.97) for the global psychological distress score; and validity has been confirmed^72^. As the factorial validity for the subscales is debated, the present study only used the Global Global Severity Index which has proven as a valid indicator of psychological distress^73^.

#### Structured Clinical Interview for DSM-IV (SCID-II)

The SCID-II assesses personality disorders according to DSM-IV in a two-tiered procedure. First a 119-item self-report screening questionnaire, which uses a Yes/No response format, asks for each of the diagnostic criteria of all personality disorders listed within DSM-IV^74^. Second, all personality disorders for which respondents endorsed sufficient criteria for a specific diagnosis are carfully evaluated by an interviewer in order to assign a formal diagnosis^75^. A modified version of the SCID Screen questionnaire resulted in a correlation of 0.84 between the number of criteria fulfilled in the SCID II interview and in the questionnaire. After adjusting the cut‐off level for diagnosis, the frequency of personality disorders found by the SCID screen questionnaire or the interview was almost the same with 58% and 54%, respectively; the overall kappa was 0.78^76^. We only used the questionnaire data in the present study which does not allow formal diagnoses. The self-defeating personality disorder is represented by 7 items, the dependent personality disorder by 8 items, the obsessive-compulsive personality disorder by 9 items, the negativistic personality disorder by 8 items, the depressive personality disorder by 8 items, the paranoid personality disorder by 9 items, the schizotypal personality disorder by 9 items, the schizoid personality disorder by 9 items, the histrionic personality disorder by 7 items, the narcissistic personality disorder by 16 items, the borderline personality disorder by 14 items, and the antisocial personality disorder by 15 items. We chose the variables contributing significantly to separating the data into clusters and compared the resulting groups regarding the number of “yes” answers in each personality disorder being aware that we were not able to diagnose the specific personality disorder on an individual basis.

#### Amsterdam Short-Term Memory Test (ASTM)

The ASTM measures negative response bias and insufficient motivation in psychological examinations^77^. It is presented as a test of short-term memory and attention. Five semantically related words are shown for 8 seconds. They should be read aloud and remembered. Then, a simple arithmetic problem is given. Afterward, another five words are presented, and the subject is required to identify the three words that were previously shown. The score for the 30 tasks totals a maximum of 90 points. The reliability of the test is satisfactory. The internal consistency in different samples is around 0.90. In a sample of mixed neurological patients, test–retest correlation was 0.85 within an interval of 1 to 3 days^77^. A different study reports a reliability coefficient of ϕ = 0.92 based on the comparison of actual versus diagnosed group membership in an experimental simulation study^78^. The test also demonstrates good validity. The cutoff value for the ASTM is ≤84 points. Sensitivity for lack of motivation was 91% (in experimental simulants) and specificity was 89% (in neurological patients). Healthy controls from age 9 on master this test almost perfectly. Patients with neurological disorders, such as concussions, brain tumors, multiple sclerosis, or difficult-to-treat epilepsy, rarely have difficulties in handling this test, provided they do not have serious cognitive deficits. The test has been shown to identify ADHD patients with severe attention impairments^59^.

#### Quantified Behavior Test Plus (Qb+)

The Qb+ is a CPT measuring sustained attention with a 1-back working memory task (recall of the same object in shape and color; see description above) combined with a simultaneous high-resolution motion tracking system. It separately assesses hyperactivity, inattention, and impulsivity with nine parameters and takes 15 to 20 minutes. Presented stimuli are a blue circle, a blue square, a red circle, and a red square. A response key is to be pressed when two identical stimuli are shown in succession. The task requires stimulus information to be maintained in working memory until the next stimulus is presented and a matching process can be done^79^. The ratio of target to nontarget stimuli is 25:75. During performance of the CPT, the movements of the participant are recorded with an infrared camera tracking a reflective marker attached to a headband worn by the participant. The infrared camera is placed about 1 m away from the participant, who is sitting in front of a computer screen. Participants are seated on a chair with back support but no armrest, to assure that they sit comfortably during testing, but do not adopt a reclining posture. Participants’ activities during the test are recorded by reading the coordinates (X and Y) of the headband marker. The position of the marker is sampled 50 times per second, with a spatial resolution of 1/27 mm per camera unit. Normative data have been gathered from 1,307 individuals between 6 and 60 years of age for both versions of the test (QbTest 6–12 and Qb+) with an even age and gender distribution^80^. *Q* scores are derived for hyperactivity, inattention, and impulsivity. They are interpreted similar to *Z* scores with a mean of 0 and a standard deviation of 1. A *Q* score ≥1.5 is regarded as an atypical result.

### Statistical analyses

Cluster analysis is an iterative process. We therefore first performed k-means cluster analyses generalized to all scales of measurement with squared Euclidean distances^43^. The k-means procedure as a person-centered approach identifies relatively homogeneous subgroups while maximizing the variability between clusters. Calculations were made with ALMO 15 (http://www.almo-statistik.de), which includes a k-means algorithm able to handle the different scaling of our variables and the large sample size^43^. This program proposes the optimal number of clusters and provides statistical measures to evaluate the appropriateness of several cluster solutions (F value, partial η^2^). Partial η^2^ represents the effect size in a general linear model (GLM). It is an omnibus effect size when examining the cluster solution as a whole and a partial η^2^ when examining the contribution of single variables to the cluster solution. η^2^ of 0.01 can be regarded as small, 0.06 as medium, and 0.14 as large^45,81^. Variables were first examined regarding their importance for cluster formation as -to the best of our knowledge- this is not available in any R package. We substantiated our analyses in R (https://cran.r-project.org/) as several R packages offer more detailed options. We applied R packages clValid^82^ and NbClust^83^ to determine the best clustering algorithm and the optimal number of clusters^46^. Variables not used for classification further characterized the two clusters on a descriptive level. If standard deviation was close to the mean, Huber’s M estimators were also listed. Differences between the two clusters on metric variables were evaluated by Welch tests which were shown to be robust against violations of normality and homogeneity of variance^84^ and by using effect size Cohen’s d with 0.2 showing a small, 0.5. a medium, and 0.8 a large effect^47^. In case of multiple testing, p values were adjusted by Bonferroni correction^85^. Priority should be given to the analysis of effect sizes as there is a critical debate over Null Hypothesis Significance Testing (NHST) and its resulting p values^86,87^.

Categorical variables were analysed by χ^2^ -Tests and effect size Cramér V of which a value ≥0.40 signals a large effect^47,79^, and by Fisher’s Exact Tests.

### Ethics committee

The study was approved by the Ethics Committee of the Department of Psychology at Philipps University Marburg, Germany.

## Data Availability

The data are available upon request.
